# A Case of Infiltrative Cardiomyopathy With Refractory Pericardial Effusion Successfully Treated With Empiric Tafamidis

**DOI:** 10.7759/cureus.48365

**Published:** 2023-11-06

**Authors:** Shudipan Chakraborty, Hafez Golzarian, Harsharn Grewal, Hemindermeet Singh

**Affiliations:** 1 Internal Medicine, Mercy Health St. Vincent Medical Center, Toledo, USA; 2 Internal Medicine, Mercy Health St. Rita’s Medical Center, Lima, USA; 3 Interventional Cardiology, Mercy Health St. Vincent Medical Center, Toledo, USA

**Keywords:** case report, atrial flutter, tafamidis, amyloidosis, pericardial effusion, transthyretin

## Abstract

Transthyretin amyloid cardiomyopathy (ATTR-CM) is a rare but fatal systemic infiltrative disease with a challenging course of both diagnosis and management. Definitive diagnosis of such rare infiltrative diseases is not feasible for most centers around the world, often leading to a delay in treatment in these patients. We present a case of suspected ATTR-CM manifesting with recurrent decompensated heart failure, tachyarrhythmias, and recurrent pericardial effusion refractory to several lines of treatment. Eventually, the patient had an excellent response to tafamidis therapy, which was initiated empirically in the absence of a definitive diagnosis. Our case elucidates the challenges of treating this rare disease and the potential effectiveness of initiating newer agents such as tafamidis sooner rather than later in the clinical course.

## Introduction

Transthyretin amyloid cardiomyopathy (ATTR-CM) is a rare but heavily underdiagnosed systemic infiltrative disease caused by pathogenic mutations, which results in destabilization and dissociation of the transthyretin tetramer into monomers that deposit in the extracellular space of the myocardium. The diffuse accumulation of these amyloid fibrils leads to conduction abnormalities manifesting as life-threatening overt tachyarrhythmias and high-degree atrioventricular blocks [1,2]. Recent novel agents, such as tafamidis (Pfizer, Manhattan, New York City), play a pivotal role in stabilizing these unstable transthyretin proteins. The path to definitive diagnosis of this disease, unfortunately, requires advanced imaging and endomyocardial biopsy, neither of which are available at most centers. Because of the rarity, nonspecific manifestations, and diagnostic barriers, it has conventionally been very difficult to treat these patients in a timely fashion. We present the case of a patient with recurrent decompensated heart failure, pericardial effusions, and overt tachyarrhythmias who failed multiple lines of therapy. Despite lacking a definitive diagnosis, our clinical suspicion for cardiac amyloidosis was high. Thus, we empirically initiated tafamidis, which provided the patient with a resolution of all his symptoms. We elucidate the challenges associated with lacking a timely diagnosis of ATTR-CM and how clinicians can potentially mitigate these challenges with early empiric use of novel transthyretin-stabilizing agents such as tafamidis.

## Case presentation

A 61-year-old female presented to the emergency department with the chief complaint of shortness of breath that started two days prior. She was orthopneic and dyspneic at rest but denied chest pain, palpitations, diaphoresis, lightheadedness, or cough. Vitals were within normal limits. Physical examination revealed bibasilar fine rales and knee-high pitting edema in her lower extremities bilaterally. Lab workup was significant for an elevated B-type brain natriuretic peptide (Pro-BNP) level of 6000 pg/mL coinciding with a chest radiograph revealing bilateral pleural effusions with interstitial edema. Serial troponins were negative. A transthoracic echocardiogram (TTE) revealed a normal left ventricular ejection fraction (EF) of 55%, grade II diastolic dysfunction, and a small pericardial effusion. Classical echocardiographic findings of amyloidosis (i.e., sparkling granular appearance of the interventricular septum) were not apparent in this study. The remaining workup including serum thyroid-stimulating hormone levels, malignancy screening, and rheumatologic workup were all unremarkable. She was provided with diuresis and later discharged with outpatient follow-up.

**Figure 1:**
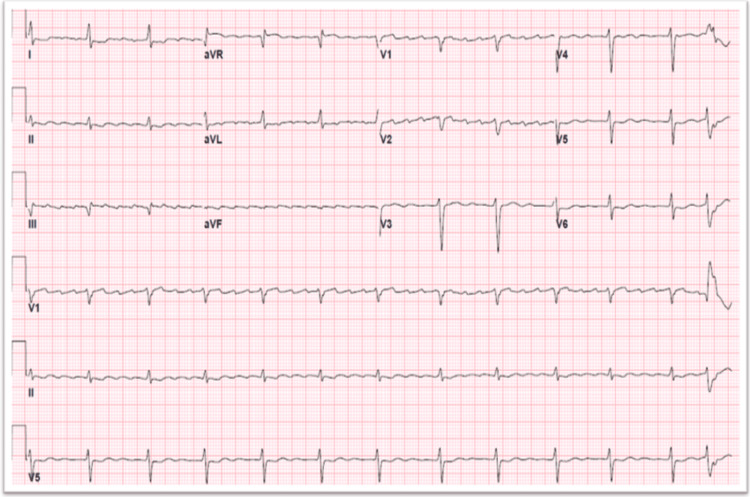
ECG revealing atrial flutter with 4:1 conduction, low voltage complexes, and signs of ischemia in lateral leads.

She returned six months later with a recurrence of the same symptoms despite maintaining strict compliance with her regimen. Her diuretic dose was doubled, spironolactone was added, and her beta blocker dosing was optimized. She was then discharged by day three of hospitalization. Three months later, she presented again with decompensated heart failure, this time with atrial flutter and new low-voltage complexes on electrocardiography (Figure 1).

**Figure 2:**
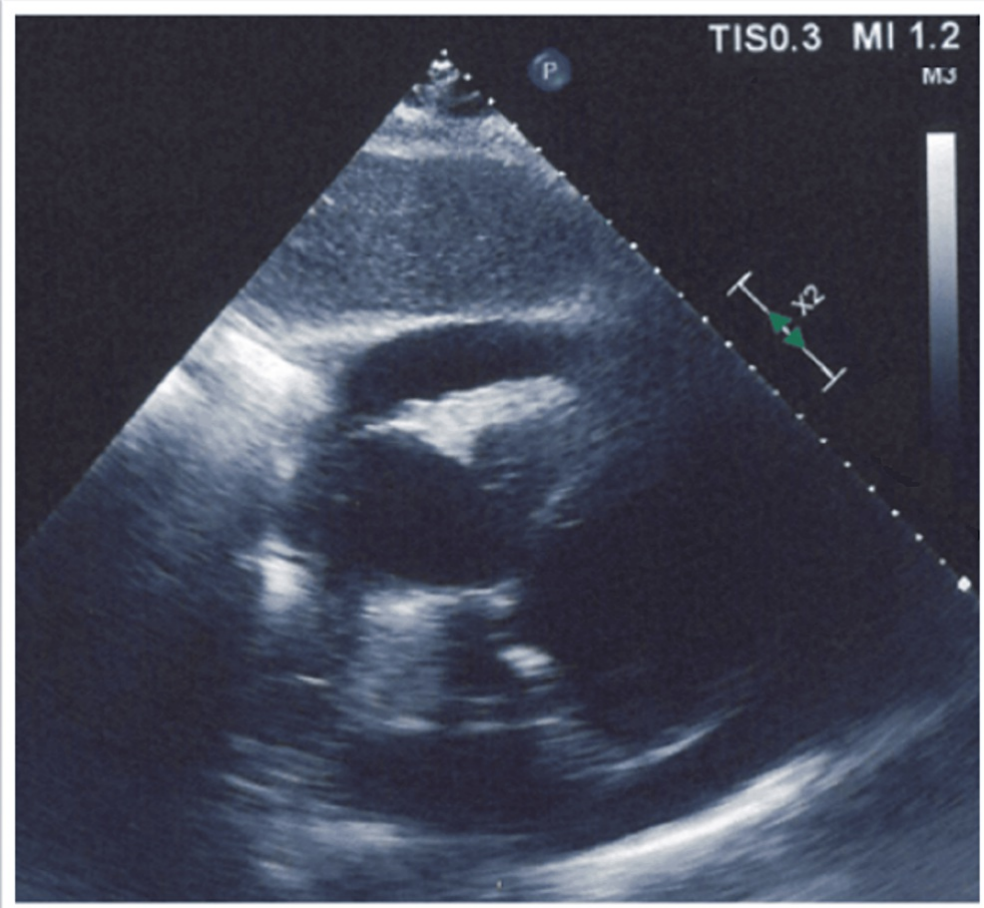
Transthoracic echocardiography revealing a moderate-large pericardial effusion.

A repeat TTE revealed a moderate-large pericardial effusion (Figure 2).

**Figure 3:**
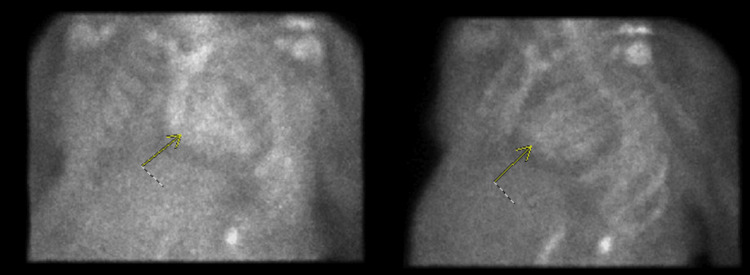
Technetium 99 M pyrophosphate-derived nuclear medicine amyloid scan was then performed, which showed Grade 3 Perugini uptake, suggestive of cardiac amyloidosis.

Pericardiocentesis of a total of 1.2 L of fluid was performed. Fluid analysis revealed a lactate dehydrogenase level of 118 U/L, glucose of 99 mg/dL, red blood cell count of 5,250 cells/uL, lymphocyte count of 36%, neutrophil count of 4%, pH of 8, and total protein of 3.9 g/dL. Given her refractory disease process, recurrent admissions, and progression of pericardial effusion, we proceeded with further workup for infiltrative cardiomyopathy. She was found to have an elevated Kappa/Lambda light chain ratio of 1.77. Technetium 99 M pyrophosphate-derived nuclear medicine amyloid scan (Tc-PYP) was then performed, showing Grade 3 Perugini uptake, highly suggestive of cardiac amyloidosis (Figure 3) [3].

The decision was made to empirically initiate tafamidis therapy, as our clinical suspicion for ATTR-CM was high. The patient responded well with a resolution of symptoms. Six months later, she remained fully asymptomatic and has been able to remain in a fully stable and compensated state.

## Discussion

Cardiac amyloidosis may be classified as systemic, localized, or hereditary. The most common subtype of amyloidosis is light-chain amyloidosis, which falls under systemic disease. Approximately 2,200 cases of light chain amyloidosis are diagnosed annually in the US, with a prevalence of only 40.5 per million, making it one of the rarest and most underdiagnosed cardiomyopathies [4]. Approximately 70% of these systemic cases manifest with renal and cardiac involvement [5]. Specifically, right ventricular failure, high-grade atrioventricular blocks, tachyarrhythmias, pericardial/pleural effusions, and orthopnea are some common manifestations of the disease process.

Our low voltage QRS complexes on electrocardiography with paradoxical left ventricular wall thickness on echocardiogram was a classic diagnostic cue to further evaluate our patient for an infiltrative process. However, in our case, the etiology of low voltage was likely compounded by the effusion. Pseudo-infarction patterns may also often be seen in both electrocardiography and echocardiography. Classic echocardiographic findings include a sparkling granular or speckled appearance of the myocardium, along with bi-atrial enlargement. Cardiac MRI has conventionally been a useful but expensive test to stratify the extent of the disease based on late gadolinium enhancement. Furthermore, gadolinium can be nephrotoxic and result in nephrogenic systemic fibrosis, especially in patients with renal involvement. Recently, Tc-PYP imaging has been proven to be a highly sensitive and reliable imaging modality to diagnose cardiac amyloidosis. A positive result indicates Grade 2 Perugini uptake (moderate cardiac uptake in relation to rib uptake) or Grade 3 (high cardiac uptake in relation to rib uptake) [6]. Despite these advancements in imaging modalities, an endomyocardial biopsy remains to be the gold standard test. Unfortunately, the vast majority of healthcare centers in the world do not have access to any of the aforementioned diagnostic tests.

In 2018, the tafamidis in the transthyretin cardiomyopathy clinical trial showed that in patients with ATTR-CM, tafamidis was associated with reductions in all-cause mortality and cardiovascular-related hospitalizations [7]. Moreover, it significantly prolonged patients’ long-term functional capacity and quality of life compared to the placebo. Most importantly, tafamidis was generally safe and well-tolerated throughout the study. Since its FDA approval, tafamidis has rapidly become one of the most commonly utilized pharmacological therapies in patients with both wild-type and hereditary ATTR-CM. It is dosed at 80 mg orally once daily. It works as a transthyretin stabilizer that selectively binds to it at the thyroxine binding sites and slows down the dissociation from its tetrameric form into monomers, which is the rate-limiting step in the amyloidogenic process. The drug is expected to have a greater benefit when initiated early in the disease course [8]. The hindrance clinicians have always faced is the inability to prove timely but proper diagnosis. We believe this is largely because of both the rarity of the disease itself as well as the inaccessibility to the guideline-recommended diagnostic tests such as cardiac MRI or biopsy. Given the excellent safety profile, initiating such treatments empirically in the setting of a strong clinical suspicion for ATTR-CM may actually provide long-term benefits to these patients.

## Conclusions

Our case elucidates the challenges in diagnosing and managing patients with systemic amyloidosis in a timely fashion, suggesting that a definitive diagnosis may not be necessary to empirically initiate tafamidis in the presence of high clinical suspicion. In our case, tafamidis has been effective in preventing recurrent pericardial effusion along with cardiac conduction abnormalities like atrial fibrillation. However, large-scale studies are necessary to further support these claims and to formulate a proper guideline for initiating tafamidis in patients with systemic amyloidosis.
